# Time to act sustainably: Why can’t we wait any longer?

**DOI:** 10.1590/0034-7167-2022-0813

**Published:** 2023-11-10

**Authors:** Patrícia de Oliveira Furukawa, Isabel Cristina Kowal Olm Cunha, Mavilde da Luz Gonçalves Pedreira

**Affiliations:** I Universidade Federal de São Paulo. São Paulo, São Paulo, Brazil

**Keywords:** Nursing, Health Services, Environment, Climate Change, Sustainable Development, Enfermería, Servicios de Salud, Ambiente, Cambio Climático, Desarrollo Sostenible, Enfermagem, Serviços de Saúde, Meio Ambiente, Mudança Climática, Desenvolvimento Sustentável

## Abstract

**Objectives:**

to raise reflections on the need for health services and professionals to implement sustainable actions, aiming at their own survival and that of the planet.

**Methods:**

reflective essay based on international reports regarding the impact of climate change on people’s health and the role of institutions in this context.

**Results:**

the article focused on three fundamentals: climate change continues to be a threat to the health and well-being of all beings on Earth; the institutions that should contribute to health are great agents of contamination of the environment and emission of gases that aggravate the greenhouse effect; and there are several benefits for health institutions to act sustainably.

**Final Considerations:**

we cannot wait any longer; we must develop policies and management models aimed at environmentally responsible, economically viable, and socially more collaborative healthcare.

## RESUMO

**Objetivos:**

suscitar reflexões acerca da necessidade de os serviços e profissionais de saúde implementarem ações sustentáveis visando à própria sobrevivência e do planeta.

**Métodos:**

ensaio reflexivo embasado em relatórios internacionais com relação ao impacto das mudanças climáticas sobre a saúde das pessoas e ao papel das instituições nesse contexto.

**Resultados:**

são enfocados três fundamentos: as mudanças climáticas continuam a ser uma ameaça para a saúde e bem-estar de todos os seres da Terra; as instituições que deveriam contribuir para a saúde são grandes agentes de contaminação do meio ambiente e de emissão dos gases que agravam o efeito estufa; e há vários benefícios para as instituições de saúde agirem de maneira sustentável.

**Considerações Finais:**

não podemos esperar mais; temos de desenvolver políticas e modelos de gestão voltados à uma assistência à saúde ambientalmente responsável, economicamente viável e socialmente mais colaborativa.

## RESUMEN

**Objetivos:**

suscitar las reflexiones acerca de la necesidad de los servicios y profesionales de salud implementar acciones sostenibles objetivando a la propia supervivencia y del planeta.

**Métodos:**

ensayo reflexivo basado en informes internacionales con relación al impacto de los cambios climáticos sobre la salud de las personas y al rol de las instituciones en eso contexto.

**Resultados:**

están enfocados tres fundamentos: los cambios climáticos continúan a ser una amenaza para la salud y bienestar de todos los seres de la Tierra; las instituciones que deberían contribuir para la salud son grandes agentes de contaminación del ambiente y de emisión de los gases que agravan el efecto invernadero; y hay diversos beneficios para las instituciones de salud actuaren de manera sostenible.

**Consideraciones Finales:**

no podemos esperar más; tenemos de desarrollar políticas y modelos de gestión vueltos a una asistencia a la salud medioambiental responsable, económicamente viable y socialmente más colaborativa.

## INTRODUCTION

We are experiencing climate change in recent times that affects all living things on the planet. These changes are related, above all, to the increase in the Earth’s temperature, due, in large part, to the excessive emission of gases such as carbon dioxide (CO_2_), compromising the greenhouse effect phenomenon^(1)^.

Although other factors alter the energy balance of the climate system, causing global warming, according to the Intergovernmental Panel on Climate Change (IPCC), an intergovernmental body of the United Nations Environment Program (UNEP), the principal cause of present warming is, with a remarkably high degree of certainty, the emission of gases resulting from various human activities that aggravate the greenhouse effect, partially masked by aerosol cooling. Human influence has warmed the planet at an unprecedented rate^(2)^.

The global surface temperature has risen faster since 1970 than in any other 50-year period of the past two thousand years. Human-induced climate change is resulting in many weather extremes in all regions of the globe, with an increase in the frequency of heat waves, droughts in several places on the planet simultaneously, fires that are difficult to control, and areas of increasingly large floods, and other consequences^(2)^.

One of the key findings of the IPCC’s 6^th^ Assessment Report (AR6) was that global surface temperatures will continue to rise until at least the middle of this century under all emissions scenarios considered. Global warming of 1.5 °C and 2.0 °C will be exceeded during the 21^st^ century unless there are deep reductions in CO emissions_2_ and other greenhouse gases in the coming decades^(2)^.

Sustainable development is the ability to meet the needs of the current generation without compromising the capacity of future generations, aligning economic growth and social demands with environmental issues^(3)^. It has been, until now, the main viable alternative, if we do not want to perpetuate irreversible changes in the climate, which compromise our survival and that of other living beings on the planet.

The First Global Initiative in this direction occurred in 1972 at the Stockholm Conference, followed by several others, such as the First World Climate Conference in 1979, the Brundtland Commission in 1983, the United Nations Conference on Environment and Development in 1992, which resulted in Agenda 21, the United Nations Convention on Climate Change, with the Kyoto Protocol in 1997, and the United Nations General Assembly (UN) in 2015, which originated the 2030 Agenda. The latter is a global compact to eradicate poverty, protect the planet and ensure that people achieve peace and prosperity through 17 sustainable development goals by the year 2030.

However, 50 years have passed since the first initiative aimed at sustainable development, and we are still moving slowly in terms of concrete actions, especially in health organizations, since they are significant contributors to the carbon footprint^(4)^.

## OBJECTIVES

To raise reflections and discussions on the need for health services and professionals to conduct sustainable actions, aiming at their own survival and that of the planet.

## METHODS

Reflective test based on the latest assessment report of the Intergovernmental Panel on Climate Change (IPCC - AR6)^(2)^, an intergovernmental body of the United Nations Environment Programme (UNEP) 2021; and The Lancet Countdown Report^(5)^, published in 2022. These documents represent the consensus of the main research leaders of academic institutions and UN agencies regarding the impact of climate change on people’s health and the role of institutions in this context.

## REFLECTIONS AND DISCUSSIONS

Until now, many institutions and health professionals still focus their policies and management models on the disease and related costs, disregarding their socio-environmental responsibility. However, people cannot ignore their commitment to the planet’s future any longer since this concerns everyone.

First of all, our world is in a state of urgency. Climate change continues to be a threat to the health and well-being of all living beings on Earth. Air pollution, soil contamination, food shortages, the spread of pathogens, antimicrobial resistance, and climate change impacts such as heat waves, storms, floods, droughts, and fires increase the demand for health services. The Covid-19 pandemic itself, caused by the SARS-CoV-2 coronavirus, is undeniably related to the climate crisis^(6)^.

It is a consensus among leading researchers from different organizations that extreme and more frequent weather events are increasingly damaging people’s physical and mental health, with the consequent reduction in working capacity and exacerbation of the risks of infections and disease outbreaks. These health impacts further increase the pressure on overburdened health systems^(5)^.

The problems arising from global warming mainly affect vulnerable and disadvantaged people, especially communities with geographical and income disadvantages. The impact of climate change is the new public health challenge and has become an important determinant of health^(6)^.

Secondly, health institutions themselves have a part in this. The health sector is responsible for 4.4% of global greenhouse gas emissions, which is equivalent to annual emissions from 514 coal-fired power plants^(2, 4, 7)^. Ironically, by striving to improve the health of the population and the individual, the health system contributes significantly to climate change, which ultimately affects human health^(8)^.

In health services, fossil fuels are the dominant source of emissions of gases related to climate change. The use of coal, oil, and gas to supply hospitals, travel related to health services, and the manufacture and transportation of medical products comprise 84% of all greenhouse gas emissions from the sector^(4)^.

There are more than 6,300 hospitals in Brazil, according to ANAHP (National Association of Private Hospitals). The operation of a single hospital without responsibility for environmental impacts can strongly contribute to the depletion of natural resources, to climate change, and, consequently, adverse effects on human health^(9)^. It is aggravated when we add up all the health services in the country and the world.

Third place, reducing greenhouse gas emissions benefits the institution itself. The need for actions that preserve the environment has led, within organizations, to the spread of the concept called “eco-efficiency,” which means creating more useful and valuable products and services by progressively reducing resource consumption and carbon emissions. It is a management philosophy in which companies are encouraged to seek environmental improvements that enhance, in parallel, economic benefits. It focuses on business opportunities, encouraging innovation and, therefore, growth and competitiveness^(10)^.

Sustainable practices are reducing waste based on the rational use of resources and process improvement^(11)^. A desirable side effect of many sustainability efforts is significant cost savings^(8)^.

This concept has received even greater visibility through a UN initiative that created, in 2004, the acronym ESG (Environmental, Social, and Governance) through the dissemination of the report Who Cares Wins^(12)^ in partnership with financial institutions from different countries. These institutions believe that better consideration of environmental, social, and corporate governance factors will ultimately contribute to stronger and more resilient investment markets as well as the sustainable development of societies.

The principles of this global compact between the UN and financial institutions include: 1) companies must support and respect the protection of internationally proclaimed human rights within their sphere of influence; 2) ensure their non-participation in violations of these rights; 3) companies must support freedom of association and the effective recognition of the right to collective bargaining; 4) must eliminate of all forms of forced or compulsory labor; 5) must effectively abolish child labor; 6) eliminate discrimination in employment and occupation; 7) companies must support a preventive approach to environmental challenges; 8) develop initiatives to promote greater environmental responsibility; 9) encourage the development and diffusion of environmentally sustainable/friendly; and 10) businesses must fight corruption in all its forms, including extortion and bribery^(12)^.

Currently, these principles are considered essential in risk analysis and investment decisions. Therefore, also in the view of investors, it is necessary for companies, including in the health area, to take care of the environment, have a social responsibility, and adopt good governance practices.

In the implementation and execution of their environmental management systems, health institutions can also rely on the services of certification companies (such as ISO 14001), which establish guidelines for organizations that intend to operate sustainably, adding value to the environment, the organization itself and stakeholders^(13)^.

In the hospital area, organizations that respect the environment in all aspects, starting with their construction, can also receive the title of “Green Hospital;” thus, they obtain recognition and differential in the market because the image of the institution is related to the commitment to the environment and society^(14)^.

A Green and Healthy Hospital promotes public health by continuously reducing its environmental impacts and, ultimately, eliminating its share in the burden of disease. It recognizes the relationship between human health and the environment and demonstrates this understanding through its governance, strategy, and operations. It connects local needs with its environmental actions and practices primary prevention by actively engaging in community efforts to promote environmental health, health equity, and a green economy^(14)^.

The Global Agenda for Green and Healthy Hospitals provides a framework of ten goals that connect hospitals and health systems of the world so they can function more sustainably, helping to improve public and environmental health. The guidelines relate to 1) Leadership: prioritize environmental health as a strategic imperative; 2) Chemicals: replace hazardous chemicals with safer alternatives; 3) Waste: reduce, treat, and safely dispose of healthcare waste; 4) Energy: implement energy efficiency and renewable clean energy generation; 5) Water: reduce water consumption and provide clean drinking water; 6) Transportation: improve transportation strategies for patients and staff; 7) Food: buy and offer healthy, sustainably grown food; 8) Pharmaceuticals: prescription 9) Buildings: support green and healthy hospital projects and constructions; 10) Procurement: buy safer and more sustainable products and materials^(14)^.

Some hospitals already use environmental sustainability indicators to measure the challenges and advances more objectively in incorporating practices that promote sustainable development^(9)^.

Health services can cooperate with the Sustainable Development Goals proposed by the UN through the 2030 Agenda^(15)^ in several respects. These targets are beyond the field of health and well-being and help to achieve improved nutrition and promote sustainable agriculture by selecting the food is purchased and delivered in their institutions; to ensure that the education that is inclusive and equitable, as well as to promote lifelong learning opportunities throughout life for all, and giving it to their employees; achieving gender equality and the empowerment of women, pointing out that the majority of the nursing team composed of women; to ensure the availability and sustainable management of water in their facilities; and to promote sustainable economic growth, inclusive growth and sustainable development, full and productive employment and decent work for all, and to build infrastructure, resilience, and promote the industrialization for inclusive and sustainable development and to encourage innovation, to ensure the patterns of production and consumption sustainable, select the products that you purchase; to take urgent action to combat climate change and its consequences; promoting the sustainable use of the oceans, seas, and marine resources for sustainable development, to help protect, restore and promote sustainable use of terrestrial ecosystems; promoting peaceful societies and inclusive and sustainable development, provide access to justice for all and build effective institutions, accountable and inclusive at all levels strengthen the means of implementation and revitalize the global partnership for sustainable development. Finally, many of the objectives of development are interconnected, so health organizations can and should assist in different ways and objectives.

The health sector must lead by example by becoming greener, not only reducing but also achieving zero carbon by 2030, as proposed by the UN Global Compact. It is not only possible but also necessary^(8)^. However, we need to act immediately and decisively so that we, the rational species on this planet, use our ability to think and create to reverse the very mistakes that have compromised everyone’s lives.

## FINAL CONSIDERATIONS

We already have enough scientific evidence to understand the seriousness of the situation, as well as to chart the path we need to follow at the same time it is possible to preserve the planet. As nurses in an emergency, we have a mission to save lives. This delicate moment of our survival requires concrete actions on the part of everyone, especially health professionals.

We can’t wait any longer: we have to develop management models environmentally more responsible, economically viable, and socially collaborative assistance. We have to improve our work processes, making them more sustainable and efficient.

We believe that the sum of individual attitudes with collective actions of people, institutions, and governments can promote the changes that help to transform this great challenge into learning and advancement. It will confirm our ability to do science, that is, to understand the phenomena of nature and use this understanding to implement actions aimed at the common good.
